# Comprehensive Evaluation of Hazardous Chemical Exposure Control System at a Semiconductor Manufacturing Company in South Korea

**DOI:** 10.3390/ijerph15061162

**Published:** 2018-06-03

**Authors:** Sangjun Choi, Chungsik Yoon, Seungwon Kim, Won Kim, Kwonchul Ha, Jeeyeon Jeong, Jongcheul Kim, Jungah Shin, Donguk Park

**Affiliations:** 1 Department of Occupational Health, Daegu Catholic University, Gyeongbuk 38430, Korea; junilane@gmail.com; 2 Department of Environmental Health Science and Institute of Health and Environment, Graduate School of Public Health, Seoul National University, Seoul 08826, Korea; csyoon@snu.ac.kr; 3 Department of Public Health, Keimyung University, Daegu 42601, Korea; swkim@kmu.ac.kr; 4 Wonjin Institute for Occupational and Environmental Health, Seoul 02221, Korea; gganna@hanmail.net; 5 Department of Biohealth Science, Changwon National University, Changwon 51140, Korea; kcha@changwon.ac.kr; 6 Department of Occupational and Environmental Health, Yongin University, Yongin 17092, Korea; jyjung@yongin.ac.kr; 7 Boram E&T Co.Ltd., Gwangju 61007, Korea; kjc8282275@gmail.com; 8 Occupational Lung Disease Institute, Korea Workers’ Compensation and Welfare Service, Incheon 21417, Korea; sja2014@kcomwel.or.kr; 9 Department of Environmental Health, Korea National Open University, Seoul 03087, Korea

**Keywords:** hazardous chemical, exposure control system, semiconductor, job-exposure matrix

## Abstract

The goal of this study was to evaluate the hazardous chemical exposure control system in a semiconductor manufacturing company and recommend an appropriate exposure surveillance system for hazardous agents. We reviewed compliance-based chemical exposure data compiled between 2012 and 2014 by the study company. The chemical management system, characteristics of chemical use and hazardous gas monitoring system were also investigated. We evaluated the airborne isopropyl alcohol (IPA) and acetone generally used as cleaning solvents, volatile organic compounds and metals levels using internationally recommended sampling and analytical methods. Based on the results of past working environment measurement data and of our investigation, the overall current exposure to chemicals by semiconductor workers during routine production work appears to be controlled below occupational exposure limits. About 40% of chemical products used were found to contain at least one unidentifiable trade-secret substance. There are several situations and maintenance tasks that need special attention to reduce exposure to carcinogens as much as possible. In addition, a job-exposure matrix as a tool of surveillance system that can examine the exposure and health status of semiconductor workers according to type of operation and type of job or task is recommended.

## 1. Introduction

Potentially hazardous chemicals such as metals, photoactive chemicals, organic solvents, acids, and toxic gases are used in the semiconductor industry in a wide variety of combinations and workplace settings. This industry also involves risks associated with radiation exposure, shift work, and other occupational stressors, including ergonomic issues. The complex nature of this highly competitive industry and rapidly changing technology and materials make it difficult to investigate chronic health risks, such as cancer, associated with exposures. In semiconductor wafer fabrication and packaging, workers have the potential to be exposed to a broad number of chemicals in manufacturing processes that have been changing dramatically over the years with the rapidly advancing technology applied in this industry. High energy is also extensively used. Concerns have recently been growing regarding the incessant development of chronic health effects, including cancer and rare diseases, among semiconductor workers. Although microchips are very small, the semiconductor industry uses hundreds of chemicals, many in significant quantities and many of them toxic [1,2].

Several epidemiological studies have been conducted to examine whether workers employed in wafer fab operations are subject to either increased cancer risk or mortality [3,4,5,6,7,8,9]. The results of epidemiological studies have been mixed, which may be due in part to the diverse exposure assessment methodologies applied [3,4,5,6]. In particular, concerns have been growing since around 2010 regarding whether several chronic diseases, including leukemia, are associated with jobs or tasks performed at several semiconductor companies in South Korea [10].

In 2015, a study on the exposure assessment and health effects of semiconductor workers was conducted at the request of a semiconductor manufacturing company in South Korea where the risk of chronic health problems, including cancer, had been raised by a newspaper [11] and civic group. This study consisted of three projects, including the evaluation of the exposure assessment program within this company, the evaluation of health problems, including cancer risk, and compensation for workers suffering diseases that may be associated with semiconductor manufacturing environments. This is one part of the studies that assessed exposure to major hazardous agents, and could be used to associate this with health problems and to provide useful exposure information for compensation related to occupational disease and recommend a suitable exposure surveillance system.

The specific aims of this study are to review historical chemical exposure data compiled by the company for compliance with the Korean Occupational Safety and Health Act (Korean OSHAct), to assess current exposure to major chemicals and to recommend an exposure surveillance system based on our results.

## 2. Materials and Methods

### 2.1. Overview of Study Company and Semiconductor Operations

The company we studied has operated since 1984 and manufactures chips for electronic products through the fabrication of integral circuits on silicon wafers in two factories operated separately in different cities. The sizes of integrated wafers currently being manufactured are eight (200 mm) and 12 inches (300 mm). Five- and six-inch wafers were manufactured until the end of 1997 and early 2001, respectively. As of 2015, a total of three wafer fabrication (FAB) facilities, one R&D center, and one probe test facility in plant A are manufacturing 300 mm wafers. Three fabrication facilities are in operation at plant B, two producing 300 mm wafers, one producing 200 mm wafers, as well as one chip packaging (PKG) facility.

Integrated circuits are fabricated on a silicon wafer through a succession of repetitive processes composed of four main operation groups: (1) patterning–oxidation, photolithography (PHOTO), developing, etching and stripping (ETCH); (2) junction formation–diffusion (DIFF) and ion implantation (IMP); (3) deposition–epitaxial or chemical vapor deposition (CVD), which are operated to deposit thin film (T/F); and (4) metallization–sputtering and evaporation.

Wafers are subjected to these steps multiple times in the FAB operation, as they alternately add and then selectively remove materials in layers from the surface of the wafer in order to create the different parts of the completed integrated circuit.

After the integrated circuitry (FAB operation) has been completed, wafers leave the clean room for the chip assembly operation, where the chips on each wafer are diced from the wafers, tested and packaged individually or into modules through a wide variety of techniques (solder ball paste print (SPP); solder ball attach (SBA); die attach (D/A); adhesive print (A/P); test during burn-in (TDBI); marking, visual inspection and packing (MVP); and module chip mount (MDL)) for use in electronic products [12,13,14,15]. The study company operates two chip packaging facilities.

The principles of semiconductor operations and the major health hazards generated in those operations have been comprehensively described elsewhere [16,17]. In this study, for the assessment of current workers’ exposure to chemical and physical hazardous agents we selected a FAB facility for the production of 200 mm wafers in plant B and one PKG facility in plant A. Both FAB and PKG facilities have clean rooms strictly controlling airborne particles, temperature, humidity, air change and differential pressure through top-down air flow system. In particular, all workers entering the FAB clean room are required to wear all-in-one coveralls, masks and gloves, and pass through the air shower chamber.

### 2.2. Review of Historical Working Environment Measurement Data from 2012 through 2014

We reviewed chemical exposure data from 2012 to 2014 collected biannually in compliance with mandatory working environment measurement regulations pursuant to the Korean OSHAct. The employer must periodically measure the work environment when workers are exposed to any of 189 chemical substances, noise and high temperature, as determined by the Korean OSHAct. Each survey was performed by a professional industrial hygiene laboratory designated by the Korean Ministry of Employment and Labor (MoEL). In principle, all samples should be collected by the full shift-based personal sampling method. For area samples, the sample should be measured as close as possible to the breathing zone of workers.

All the measurement data were pooled and then analyzed. Data were categorized by type of sample (personal or area), type of work (operation or maintenance), and carcinogenicity (carcinogen or non-carcinogen). Using the limit of detection (LOD), the status of numbers and percentage of samples were compared by category. In terms of carcinogenic chemicals detected, we summarized more detailed information, such as the carcinogenic group, the number of samples detected and the maximum ratio of time-weighted average (TWA) concentration to occupational exposure limit (OEL). Information on carcinogenic classification and OEL, as determined by the Korean MoEL, were used [18].

### 2.3. Chemical Management and Use

During the project, the research team held frequent meetings with the Environmental Safety and Health (ESH) team and other related personnel in order to review the ESH system at the company. The company provided us with information on how the system works and one researcher temporarily accessed the ESH web system and reviewed the system. A Microsoft Excel spreadsheet containing chemical lists and respective material safety data sheets (MSDSs) were provided by the ESH team at the company in 2015. Some chemical products on the list did not match with the MSDS provided because of the phasing out of some chemicals and/or outdated MSDS. The research team communicated several times to ascertain the accuracy of the chemical list, and this list was analyzed for this study. We confirmed the exactness of the constituents of each chemical and its contents by individually comparing the list and MSDS. We also added to the Excel spreadsheet some hazard information described in the MSDS, including carcinogen, mutagen and reproductive toxins (CMR) designation.

After confirming and complementing some hazard information in the spreadsheet, the characteristics of chemical products, including the number of chemical products and their constituents, the number of products containing trade secrets and the trade-secret content were examined.

### 2.4. Airborne Volatile Organic Compounds (VOC) and Metals

Isopropyl alcohol (IPA) and acetone, the representative cleaning solvents used in this company, were measured via adsorbent tube for several processes in both the FAB and PKG facilities. In the FAB, personal short-term sampling was mainly conducted during preventive maintenance (PM) tasks, except the PHOTO process where sampling was performed during the photo resist (PR) bottle exchange task. All samples were desorbed with carbon disulfide (CS_2_) and analyzed by gas chromatography-flame ionized detector (GC-FID, HP 5975C, Agilent Technology, Santa Clara, CA, USA). The LODs of IPA and acetone were 0.007 μg/sample and 0.011 μg/sample, respectively.

In the PKG facility, VOC constituents were sampled in several processes using Tenax sorbent tubes (Tenax^®^ TA SS, Supelco, Bellefonte, PA, USA). VOCs in the Tenax tubes were desorbed with thermal desorption apparatus (GERSTEL TDS, Mülheim an der Ruhr, Germany) and quantitatively analyzed using gas chromatography-mass spectrometer (GC-MS, HP 5975C, Agilent Technology, USA).

Airborne metals were sampled with mixed cellulose ester (MCE) membrane filters (mixed cellulose ester membrane, 5.0 μm, 225-8-01, SKC Inc., Eighty Four, PA, USA). MCE filters were dissociated with hot nitric acid/perchloric acid, and the extracted metals were analyzed with an inductively coupled plasma-mass spectrometer (ICP-MS, NexION 350, Perkin Elmer Co., Beaconsfield, UK). The LODs of As, Ag, Al, Cu, Pb and Sn were 0.87, 0.009, 0.10, 0.008, 0.005 and 0.015 μg/sample, respectively.

Total volatile organic compounds (TVOC) were also measured in real time using a PPB Rae 3000 (Model PGM 7340, RAE Systems Inc., San Jose, CA, USA) photoionization detector (PID) with a 10.6 eV gas-discharge lamp featuring a detectable range from 1 part per billion (ppb) to 10,000 parts per million (ppm). Monitoring was performed at one FAB on nine days from February to June in 2015, and at the PKG facility on three days in July 2015. At each monitoring location of the operation and maintenance areas, the monitor was positioned at the center of the working area and 1.5 m above the ground. For maintenance work, the monitor was placed adjacent to the worker’s breathing zone and as close as possible. Each PID was operated at a flow rate of 500 mL/min with a data logging period of three seconds. All PIDs were calibrated with isobutylene standard gas at a calibration laboratory (KC11-254) accredited through the Korea Laboratory Accreditation Scheme (KOLAS).

### 2.5. Evaluation of Hazardous Gas Monitoring System

Gaseous chemicals like arsenic, silane, chlorine, and fluorine are essential in a variety of semiconductor manufacturing process. When incautious handling of these chemicals in the storage, distribution, and disposal processes happens, they can be released into the workplace or the general atmosphere, potentially provoking a disaster. In order to prevent this situation, most semiconductor companies have introduced and operate a continuous gas monitoring system. The hazardous gas monitoring system built in one FAB facility in plant A, as well as data on events triggering alarms over the 10 months from October 2014 to July 2015, were reviewed. We investigated the number and location of detectors installed, the level of alarms, the cause of alarm events and the chemicals frequently triggering alarms.

### 2.6. Statistical Analysis

Past measurement data are compliance-based measurement results that are mandatory and periodically evaluated by law. However, many measurement results were not detected. Therefore, the Chi-square test was used to determine whether there was a difference in the detection rate depending on the sample type, work type, and carcinogenicity.

In order to characterize the distributions of hazardous agents by process and working area, descriptive statistics such as arithmetic mean (AM), standard deviation (SD), geometric mean (GM), geometric standard deviation (GSD), maximum (Max) and range were used. If necessary, the average levels were compared by processes and working areas at the significant level of 0.05 using ANOVA or *t*-test through the test of the distributions. All data analyses and graphs were performed using IBM SPSS software version 20.0 (IBM corporation, New York, NY, USA) and Sigma Plot software, version 12.5 (Systat Software Inc., San Jose, CA, USA), respectively. This study protocol was approved by the Institutional Review Boards of Korea National Open University (ABN01-201502-11-02). The contents of this paper were also reviewed by the subject company for business secret matters.

## 3. Results

### 3.1. Review of Historical Exposure Data Compiled from 2012 through 2014

We classified the historical exposure data based on the type of operation and job and the level of toxicity by plant (Table 1). The overall non-detection rate from bi-annual monitoring surveys was near 90%. Detection rates by category in both plants showed statistically significant differences (*p* < 0.01). Numbers of samples from maintenance work were smaller (plant A = 4628; plant B = 1205) than those from operation work (plant A = 14,830; plant B = 11,320). Samples from maintenance work in plant A were taken only in 2014. In plant B, the number of samples from maintenance work rapidly increased from 120 in 2012 to 704 in 2014.

The carcinogenic chemicals above the detection limit were arsenic (and its organic compounds), formaldehyde, nickel (insoluble inorganic compounds), hydrogen peroxide, naphthalene, nickel (metal), sulfuric acid (strong acid mist), titanium dioxide, tetrahydrofuran, and welding fumes (and dust). The maximum ratio of TWA of carcinogenic chemicals to occupational exposure limit ranged from 0.002% for titanium dioxide to 37% for sulfuric acid.

A total of 3401, 4923, and 11,134 data measurements in plant A and 3227, 3577, and 5721 in Plant B were recorded in 2012, 2013 and 2014 (data not shown in table), respectively. The percentages below the LOD level in each year were 71.1%, 81.9%, and 88.3% for plant A and 90.6%, 91.1%, and 88.1% for plant B. No chemical concentration during 2012 to 2014 exceeded half of the OELs listed in the Korean OSHAct. In plant B, the highest exposure levels to sulfuric acid, ozone, and hydrogen chloride were measured in the IMP, ETCH, and T/F processes, respectively. Process information was not readily available for plant A.

### 3.2. Chemical Management System

There were three major chemical management programs in this company: environmental safety and health quality review of newly purchased chemical products by an electronic system (called ESH Qualification), MSDS provisions including e-MSDS (electronic provision of MSDS), and a database of chemical products and their use. However, these programs and elements were not well connected with one another, and some information was unavailable. For example, the ESH team and other management team lacked information on what chemicals and what amounts were used in specific processes or tasks.

However, the ESH Qualification system via the company’s internal web was well updated in 2014, so all newly purchased items or products could be reviewed as to whether there was any possibility of their containing hazardous substances regulated by the company. In this system, a total of 676 chemical substances were prohibited from entry to the company: 28 substances regulated by the Water Quality Management Law; six substances regulated by the EU RoHS (Restriction of Hazardous Substances); one (perfluorooctanyl sulfonates, PFOS) voluntary emissions reduction material by the World Semiconductor Council; eleven substances known as severe health hazards; and 631 substances regulated by the Korean MoEL or voluntarily regulated by the company.

**Figure 1:**
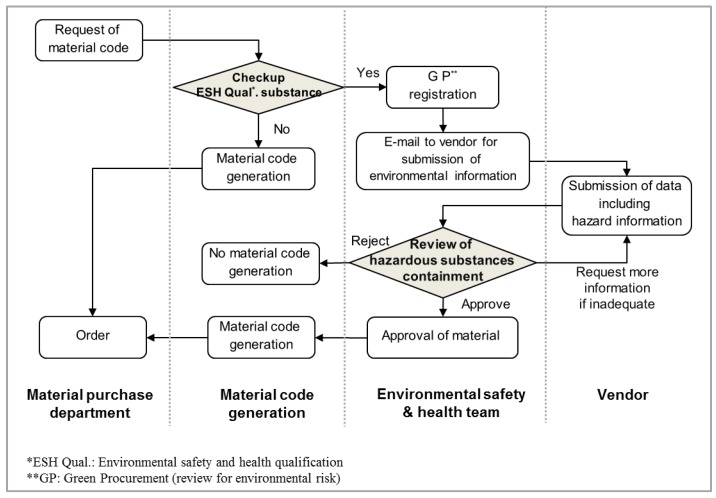
Environmental safety and health quality process for the prevention of hazardous material purchase.

Figure 1 shows how the ESH Qualification system operates. If any department in the company wishes to purchase an item, it should be enrolled in the ESH Qualification system for screening. If an item has any chemical constituent regulated by the ESH Qualification system, it should be registered with the Green Procurement (GP) process and reviewed for environmental risk. If registered in the GP process, the vendor should provide necessary hazard information via the Internet using the system. Next, the ESH team reviews all data submitted and judges whether or not a regulated substance is contained in that item. Over six months in 2015, a total of 29,000 items were entered into the ESH Qualification system, and among them, 677 materials (2.3%) were registered in the GP process. Finally, three materials (0.4%) were disallowed due to hazard.

### 3.3. General Characteristics of Chemicals Used

The general status of chemical products used, their constituents, contents of trade-secret chemicals, chemicals having OEL and chemicals under the Korean OSHact regulation are summarized in Table 2.

A total of 428 and 432 chemical products were used in plants A and B, respectively. Among these, 228 products were used simultaneously in both workplaces, or in other words, about 47% (200 products in plant A and 204 products in plant B) of chemical products were used independently, although both plants have similar FAB and PKG processes. The chemical products used in the plants consist of multi-component substances. On average, 2.6 chemicals were included in chemical products between the two plants. After removing chemical substances repeated in products, 189 and 157 individual substances were used in workplaces A and B, respectively.

About 40% (186/428 products or 44% in plant A and 168/432 products or 39% in B) of chemical products contained at least one trade-secret substance. The numbers of substances designated as trade-secret were 345 and 363 in plants A and B, so that the average numbers of trade-secret constituents per trade-secret-containing product were 1.8 and 2.1, respectively.

Fifty percent (50%) of the products in use in the plants contained constituents with an occupational exposure limit. Approximately 40% of products contained one trade-secret ingredient. Of the products in use in Plant A, 29% contained at least one ingredient requiring workplace monitoring and 24% of the products contained at least one ingredient requiring mandatory special health examinations for workers. Similar results were noted for Plant B, with 36% containing at least one ingredient requiring workplace monitoring, and 31% of products containing ingredients requiring mandatory special health examinations.

The fraction of trade-secret constituent in products varied widely from less than 1% to more than 80% (data not shown in table).

### 3.4. Airborne VOC and Metals

Airborne IPA and acetone concentrations monitored both in the FAB and PKG facilities are summarized in Table 3. In the FAB facility, in 79% of samples (31 out of 39) no IPA was detected, while for acetone the number was 87% (34 out of 39). In the PKG facility, 79% (15 out of 19 samples) and 58% (11 out of 19 samples) showed no detection. Detected airborne concentrations of IPA and acetone were at the several ppm levels and were far below the Korean OEL level for IPA (200 ppm) and for acetone (500 ppm). A relatively high measured concentration of IPA was found for the PHOTO process (29 ppm), and acetone was measured as being relatively high in the adhesive print (A/P) line or A/P cleaning room in the PKG facility, but both fell well below the Korean OEL.

**Figure 2:**
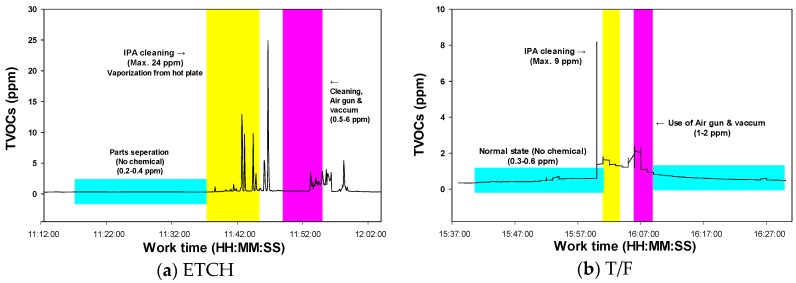

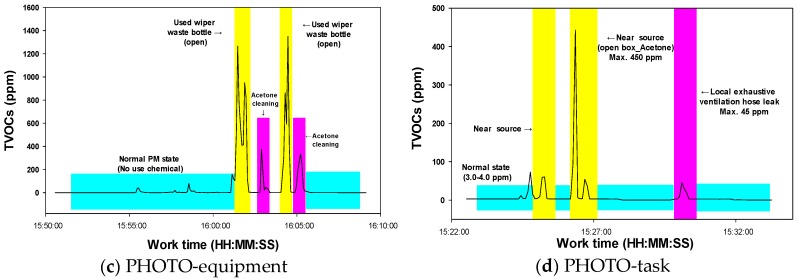
Representative examples of high total volatile organic compound (TVOC) concentrations during maintenance tasks in fabrication facility. (**a**) isopropyl alcohol (IPA) cleaning in etching process; (**b**) IPA cleaning in thin film process; (**c**) TVOC concentration measured in trashcan where acetone-soaked cloth (after cleaning) was stored among photolithography track equipment; (**d**) acetone cleaning in photolithography track breakdown maintenance task.

However, as seen in Figure 2, IPA and/or acetone concentrations could reach a very high level during short periods in a specific task, polluting the clean room with VOCs. TVOC concentrations, most of it as IPA, reached as high as 24 ppm for a short time due to the cleaning of hot metal surfaces with IPA during PM at ETCH, and afterwards air gun use also facilitated the evaporation of IPA to the level of 6 ppm, as seen in Figure 2a. A similar pattern was observed during the PM task in T/F, even though the measured TVOC concentration was lower (9 ppm) than in ETCH PM (Figure 2b). Acetone concentrations measured as TVOC reached over 1200 ppm in the open part of the trash can where acetone wiper cloth was stored, and its concentration was over 300 ppm during the task of cleaning PHOTO track equipment, as shown in Figure 2c,d. In the FAB facility, many trash cans were located without any exhaust ventilation system where wiper cloths soaked with cleaning solvents like IPA and acetone were placed after use. As a result, these solvents could easily evaporate into the cleaning room. As seen in Figure 2d, a high concentration of acetone (45 ppm) was measured at a leaky area in the flexible duct connected to a local exhaust ventilation system which was recently introduced for use during PM work.

Tenax tubes were used to measure individual VOC constituents in the PKG facility; the results are summarized in Table 4. Hexane, MIBK (Methyl Isobutyl Ketone), and butyl acetate are not presented due to their not being detected in samples despite being detected in low concentrations (ppb level) in the control sample (sampled from outdoor air inside the plant).

Concentrations of benzene, known to be a human carcinogen, were below the ppb level in several tasks, including molding (module chip mount), TDBI (test during burn-in) and testing processes, but all levels were very low compared to the Korean OEL of 1 ppm. The highest concentration was the 1.7 ppm of IPA measured in the module chip mount task, but this level was still very low compared to the Korean OEL of 200 ppm.

Toluene, xylene (ortho, meta and para) and other organic materials listed in Table 4 did not surpass outdoor control samples, with the exception of toluene in the testing and marking, visual inspection and packing (MVP) processes and IPA in all process. No level detected reached the ppm level, remaining at the ppb level instead.

Airborne metals in the PKG facility are also summarized in Table 4. As, Al, Cu, Pb and Sb levels were similar to or lower than in the control samples. Ag and Sn concentrations in solder ball attach (SBA) or solder ball paste print (SBPP) processes were higher than in the control samples due to the use of solder balls containing Ag and Sn, but the airborne concentrations (Ag 0.01–0.32 μg/m^3^; Sn 0.03–0.84 μg/m^3^) were much lower than the Korean OEL for silver (100 μg/m^3^) and tin (2000 μg/m^3^).

### 3.5. TVOC Levels in the FAB and PKG Facilities

The TVOC levels measured in the FAB facility are summarized in Table 5. In the operation areas, the PHOTO process showed the highest level for the GM concentration of TVOC at 255.2 ppb, while DIFF showed the significantly lowest level at 22.5 ppb (*p* < 0.01). The GM TVOC concentrations measured in the maintenance working area for DIFF (410.5 ppb), PHOTO (497.6 ppb) and T/F (797.6 ppb) showed significantly higher levels than the operation areas (*p* < 0.01). A maximum short-term concentration of 671 ppm was detected near the cleaning box filled with 20 L of acetone and dismantled parts from PHOTO equipment. The major maintenance tasks with the potential for short-term high exposure to TVOC were the cleaning of equipment using IPA or acetone mentioned in Figure 2 and the exchange of PR.

The distribution of TVOC concentrations measured at the PKG facility is also summarized in Table 5. The highest TVOC concentration was measured in the A/P process, with a GM of 45.7 ppb, followed by that of the test handler process with a GM of 45.2 ppb. Over the course of A/P, they used epoxy and silicon adhesives to attach dies to substrate. Next, the remaining adhesives on substrate were washed with roll papers containing acetone. A combination of these processes would have contributed to the high TVOC concentrations.

### 3.6. Hazardous Gas Monitoring Data

As shown in Table 6, 47 chemicals, 73.4% of the total gaseous chemicals used, were monitored by 1486 gas detectors built into the process equipment. A total of 101 alarm events occurring during the ten months from 2014 to 2015 were reported. The gas most frequently causing alarms was tetraethyl orthosilicate (TEOS, 43.6%), followed by difluoromethane (17.8%) and hydrogen bromide (8.9%). Most events (70.3%) occurred during preventive maintenance work using a cleaning agent, such as isopropyl alcohol, around the area of a gas detector. For 16.8% of events, the exact causes of the alarm could not be found. In terms of alarm level, 38.6% of these events were found to involve levels of chemicals two times higher than the OEL.

## 4. Discussion

Based on the results of historical exposure data and our investigation, overall current exposure to chemicals among semiconductor workers during routine production work appears to be controlled below existing or recommended standards. Routine exposures exceeding OEL were found neither among operators nor among maintenance workers. Most chemicals were below 50% of the OEL when and where working environments were well controlled (Table 1). Our results are in accordance with those reported by Marano et al. (2010), who showed that during the years 1978–2004 in the study facilities, >98% of the air samples evaluated were below the present-day OEL and over half of the samples were below the LOD [13]. They extracted approximately 60,000 sample measurements of more than 60 chemicals from company records, including bulk, wipe, and area samples, as well as both short- and long-term personal air samples, and evaluated 12,300 short-term and long-term personal air samples collected within cleanrooms only and compared them with the most current OELs. Current exposure to chemicals of operators and supervisors who manage an entire process may be lower than it was in the past, when chemicals and by-products were handled manually, since chemical handling has become more automated and enclosure of process units and gas supply lines has increased substantially.

However, there were several situations and maintenance tasks that require special attention in order to ensure that exposure to a number of chemicals, including CMR, as well as to radiation that includes extremely low-frequency magnetic fields (ELF-MF) are minimized to the degree possible to eliminate the potential for overexposure.

Particularly prior to their purchase, CMR that are known to be used intensively in semiconductor operations should be examined in terms of whether they can be eliminated or their use limited. 189 chemical constituents were included in 428 chemical products in plant A and 157 constituents were in 432 chemical products in plant B. The characteristics of most by-products generated during operations cannot be identified, which makes them difficult to assess. Although exposure levels were below half of OELs, the possibility of potential exposure to confirmed human carcinogens such as arsenic and inorganic compounds, formaldehyde, nickel (insoluble inorganic compounds) and strong sulfuric acid mist cannot be ruled out through the review of historical work environment monitoring data (Table 1).

Recently, the company put in place a chemical management system as a response to the domestic and international ESH issue (Figure 1). For example, the ESH Qualification system has recently been updated and used as an effective tool to screen out designated hazardous substance before their entry to the workplace. In the past, this system was focused simply on environmental aspects, so workplace health and safety risks were not well controlled. Still, although the staffs for the environment, safety, and health are grouped into a single ESH team, their coordination has not been well established. As mentioned above in the results section on chemical use, about 40% of chemical products contain trade-secret substances, meaning that both suppliers and the company ESH team have no tools to regulate these substances. In response, the company recently began to ask suppliers to provide a certificate of not containing CMR or other severe hazardous constituent in order to ensure that suppliers of chemicals do not abuse business confidentiality to conceal toxic chemicals, including CMR. The research team has suggested a so-called ‘trade-secret substance management program’ to reduce both the number and amount of such products and their trade-secret constituents.

In terms of chemical use, more than 200 chemical products were used independently in each plant, while 228 products were used between the two plants combined (Table 2). The reason for this is that, although these plants have both FAB and PKG facilities, the detailed processes and output products are not identical. For example, plant A alone has a copper process which uses copper for interconnections in the metallization layer in place of aluminum. However, one thing to note is that there has been no meeting or drive to harmonize the use of chemicals between the two plants.

Among the 189 and 157 chemical constituents used in plants A and B, respectively, 128 of them were used in both plants. Fifty-six chemical constituents were used only in plant A, and 24 were used exclusively in plant B. Therefore, after eliminating repetitive counting, a total of 208 chemicals excluding trade secrets were used by this company. We also found that less than 40% of chemical products could be monitored in the workplace, and 30% of chemical products could be monitored by special medical examinations when they were used. Only 44 among 189 identifiable constituents in plant A and 46 among 157 constituents in plant B have OELs. The other portion was not regulated by the Korean OSHAct. Among these, some chemical products might consist of non-hazardous substances, some have hazardous components, and some lack sufficient hazard information. One thing to note is that roughly 40% of chemical products contained trade-secret constituents about which we have no information on the chemical characteristics, including hazards. In addition, some products contain a large fraction of trade-secret constituents (Table 2). In particular, more than 90% of chemical products used in photolithography (known as photoresist) contained at least one—and on average more than two—trade-secret ingredients per product. Therefore, this unknown realm attributable to trade-secret constituents should be considered in the future in order to ascertain safe chemical use in the workplace.

Potential exposure to chemical or physical hazardous agents during maintenance or operations remains similar to that in the past despite process automation, because equipment, machines and materials must be periodically maintained or repaired through manual handling. Upset conditions during maintenance work could not be evaluated. These conditions feature a higher potential for worker overexposure than normal operating conditions, so special attention should be given to minimizing exposure. It is very difficult to assess exposure to hazardous agents in maintenance work during irregular maintenance efforts. Based on the literature review, little exposure data has been assessed during maintenance work. In particular, irregular and warranty maintenance work has been reported. Most exposure assessment was focused on TWA levels taken during regular full-shift operation work [19]. Exposure levels from maintenance work may vary considerably depending on the cleaning frequency, the contamination level of the equipment for cleaning, maintenance area, presence and efficiency of engineering controls, dust handling techniques and so on. Exhaust ventilation and enclosure were not sufficient to reduce exposure to dopant gases generated during maintenance activities. Maintenance personnel could be at risk of exposure because the performance of these activities frequently entails workers bypassing or disabling engineering controls [19]. In this study, we also confirmed arsenic exposure possibility of workers during preventive maintenance of ion implanters. The more detailed information can be found from another article published [20].

There are several operations or jobs in semiconductor operations where peak exposure should be evaluated. Hallock et al. (1993) assessed task and peak exposure to solvents including 2-ethoxyethylacetate, n-butyl acetate, xylene, isopropanol, acetone, and propylene glycol monomethyl ether acetate used in microelectronic fabrication clean rooms through both charcoal tubes and direct reading instrumentation. All measured samples were considerably below OEL [21]. We found that peak exposure to VOC (Figure 2) and ELF-MF during either maintenance work or operation can occur (not presented in this article). More detailed characteristics of ELF-MF exposure can be found in another article published [22]. Workers at the semiconductor workplace are exposed to various chemicals and ELF-MF simultaneously. In a Swedish study in 2002, it was reported that the interaction effect of an ELF-MF with a cancer-causing chemical exposure could increase the incidence of brain tumors [23]. In this study, it was confirmed that cancer–causing chemicals were detected in the air, although the concentration was low. Therefore, in order to prevent cancer in future, it is also necessary to manage the exposure considering the interactions between carcinogenic chemicals and ELF-MF.

## 5. Conclusions

The semiconductor plant we studied lacks a surveillance system with the capacity to efficiently examine the exposure and health status of semiconductor workers according to type of operation and type of job or task. Currently, there is no way to examine by operation how chemicals with high toxicity, including CMR, are used. The type and amount of chemicals used by operation cannot be recognized. The past exposure profile of not only current, but also former workers cannot be traced. It is very difficult to identify not only all of the chemicals used, but also the byproducts produced among processes. There are insufficient recordings to estimate past exposure to operation and job which can be used to associate the development of health effects, including cancers. Historical exposure data, an inventory of chemicals used in operations and gas detector systems were evaluated as not having been appropriately managed for the assessment of exposure to hazardous agents generated in semiconductor operations. There is an urgent need to establish a surveillance system to trace exposure to hazardous agents and possible health risks among semiconductor workers. Because of difficulty and inefficiency in finding peak exposure by current workplace monitoring system, chronic nature of most occupational disease, the many unknown of hazards including many chemicals with business secret and diseases and its causal association, we suggested the ESH team of the company to develop an exposure and health surveillance system that could efficiently integrate the job-exposure matrix (JEM), which reflects long-term exposure and individual worker’s health examination data. Relative risk can be assessed by the combination of process and job semiconductor workers are involved in based on JEM. In general, semiconductor workers who perform irregular maintenance work can be prioritized and should be monitored regularly and systematically. We recommend an exposure surveillance system based on JEM. This surveillance system is currently under construction for the recording of exposure profiles for all workers, including location, operation and jobs involved.

## Figures and Tables

**Table 1:** Summary of the review of work environment monitoring results from 2012 to 2014.

Plant	Classification		Number of Samples (%)					
			Detection		Non-Detection		Overall	
A	Type of sample *	Personal	3053	(16.6)	15,344	(83.4)	18,397	(100)
		Area	123	(11.6)	938	(88.4)	1061	(100)
	Type of work *	Operation	2730	(18.4)	12,100	(81.6)	14,830	(100)
		Maintenance	446	(9.6)	4182	(90.4)	4628	(100)
	Carcinogenicity *	Carcinogen	407 ^1^	(11.4)	3150	(88.6)	3557	(100)
		Non-carcinogen	2769	(17.4)	13,132	(82.6)	15,901	(100)
		Total	3176	(16.3)	16,282	(83.7)	19,458	(100)
B	Type of sample *	Personal	712	(7.7)	8554	(92.3)	9266	(100)
		Area	588	(18.0)	2671	(82.0)	3259	(100)
	Type of work *	Operation	1107	(9.8)	10,213	(90.2)	11,320	(100)
		Maintenance	193	(16.0)	1012	(84.0)	1205	(100)
	Carcinogenicity *	Carcinogen	461 ^2^	(26.3)	1289	(73.7)	1750	(100)
		Non-carcinogen	839	(7.8)	9936	(92.2)	10,775	(100)
		Total	1300	(10.4)	11,225	(89.6)	12,525	(100)

* *p* < 0.01 (Chi^2^-test); ^1^ Chemical name (carcinogenicity class, number of samples, maximum ratio of TWA to occupational exposure limit): Arsenic & inorganic compounds (1A, 22, 0.08), Formaldehyde (1A, 313, 0.06), Nickel (insoluble inorganic compounds) (1A, 2, 0.02), Hydrogen peroxide (2, 1, 0.01), Naphthalene (2, 6, 0.0004), Nickel (metal) (2, 10, 0.0008), Sulfuric acid (strong acid mist) (1A, 8, 0.042), Titanium dioxide (2, 26, 0.00002), Tetrahydrofuran (2, 1, 0.1), Welding fumes and dust (2, 18, 0.026); ^2^ Chemical name (carcinogenicity class, number of samples, maximum ratio of TWA to occupational exposure limit): Formaldehyde (1A, 52, 0.07), Nickel (insoluble inorganic compounds) (1A, 6, 0.003), Sulfuric acid (strong acid mist) (1A, 120, 0.37), Hydrogen peroxide (2, 282, 0.21), Titanium dioxide (2, 1, 0.0012).

**Table 2:** Characteristics of chemical products and constituents used in plants A and B.

Items	Classification	Plant A, N (%)	Plant B, N (%)
Products	Overall	428 (100.0)	432 (100.0)
	Containing at least one trade-secret ingredient	186 (43.5)	168 (38.9)
	Containing at least one ingredient with an occupational exposure limit	205 (47.9)	240 (55.6)
	Containing at least one ingredient for mandatory working environment measurement	124 (29.0)	157 (36.3)
	Containing at least one ingredient for mandatory special health examination	102 (23.8)	133 (30.8)
Constituents	Overall	534 (100.0)	520 (100.0)
	Identifiable chemicals without multiple counting	189 (35.4)	157 (30.2)
	Unidentifiable trade-secret chemicals	345 (64.6)	363 (69.8)
	Chemicals with occupational exposure limits	44 (8.2)	46 (8.8)
	Chemicals for mandatory working environment measurement	124 (23.2)	157 (30.2)
	Chemicals for mandatory special health examination	102 (19.1)	133 (25.6)

**Table 3:** Acetone and isopropyl alcohol concentrations in fabrication and package plants.

Facility	Process	Isopropyl Alcohol			Acetone		
		Number of Samples	Number of No Detection Samples	Range (ppm)	Number of Samples	Number of No Detection Samples	Range (ppm)
Fabrication	PHOTO	17	15	0.08–28.99	17	17	
	DIFF	7	4	0.26–0.81	7	4	0.02–0.15
	CMP	4	3	0.26	4	4	
	T/F	4	4		4	3	0.06
	IMP	3	1	0.25–0.26	3	2	0.01
	ETCH	4	4		4	4	
Package	A/P	3	3		3	0	6.23–9.07
	A/P cleaning room	1	1		1	0	9.66
	D/A	4	4		4	0	0.96–4.32
	Chip mounting	3	0	1.24–2.37	3	3	
	TDBI	7	7		7	7	
	SPP	1	0	8.28	1	1	

PHOTO: photolithography, DIFF: diffusion, CMP: chemical mechanical polishing, T/F: thin film, IMP: ion implantation, ETCH: etching, A/P: adhesive print, TDBI: test during burn-in, SPP: solder ball paste print.

**Table 4:** Airborne volatile organic compounds (VOCs) and metal concentrations in package and test processes.

Agents	Chemicals	MDL	SBA	SPP	TDBI	TEST	Test & MVP	Control ^†^
VOCs (ppb)	Number of samples	5	0	0	3	1	3	1
	Benzene	0.14–0.34			0.06–0.18	0.22	ND-0.15	ND
	Toluene	2.02–3.53			1.98–3.38	3.26	2.6–163.7	3.78
	Ethylbenzene	0.29–0.48			0.26–0.33	0.08	0.33–0.76	1.04
	p-Xylene	0.38–0.60			0.34–0.48	0.09	0.46–0.51	0.59
	m-Xylene	0.01–0.10			ND-0.12	ND	0.04–0.44	0.12
	o-Xylene	0.15–0.28			0.12–0.23	0.02	0.17–0.64	0.19
	Isopropyl alcohol	0.94–1720			5.80–11.9	ND	11.7–23.5	ND
	Methyl ethyl ketone	0.54–1.53			0.63–0.96	0.48	0.89–1.26	ND
	Tetrachloroethylene	ND–21.5			0.19–0.40	ND	0.35–0.91	ND
	1-propoxy-2-propanol	ND-58.8			ND	ND	ND	ND
	Styrene	ND-0.45			ND-0.25	0.18	0.36–0.55	ND
	Benzaldehyde	0.75–1.02			0.48–0.55	0.6	0.70–1.10	1.02
	2-Ethyl-1-hexanol	0.99–4.83			0.63–1.32	0.29	0.65–1.40	ND
Metals (μg/m^3^)	Number of samples	0	3	3	3	0	0	4
	As		0.08–0.16	ND	ND			ND-0.12
	Ag		0.01–0.32	0.01-0.26	ND-0.05			ND-0.04
	Al		4.98–7.25	ND	ND-0.06			ND-26.2
	Cu		0.93–2.07	ND-0.38	ND-0.64			ND-2.04
	Pb		0.066-0.068	ND-0.06	ND-0.01			0.01-0.10
	Sb		ND	ND	ND			ND
	Sn		0.26–0.61	0.03-0.84	ND-1.17			0.03-0.22

MDL: module chip mount, SBA: solder ball attach, SPP: solder ball paste print, TDBI: test during burn-in, TEST: electrical test, MVP: marking, visual inspection and packing; ND: not detected, ^†^ Control: outdoor air inside plant.

**Table 5:** Total volatile organic compound concentrations by process in semiconductor fabrication and package facilities.

Facility	Process	Task	Total Volatile Organic Compounds					
			N	AM, ppb	SD, ppb	GM, ppb	GSD	Max, ppm
Fabrication	CMP	Maintenance	302	336.2	3544.0	36.1	4.1	54.9
	DIFF **	Operation	388	24.1	8.6	22.5	1.5	0.038
		Maintenance	3287	939.4	2527.4	410.5	2.7	34.5
	ETCH	Operation	742	194.9	48.1	189.3	1.3	0.319
		Maintenance	2838	326.9	1182.9	184.8	2.5	30.7
	IMP	Operation	228	200.5	20.3	199.5	1.1	0.292
		Maintenance	8342	229.9	337.6	199.6	1.8	16.4
	PHOTO **	Operation	1319	274.4	126.2	255.2	1.4	0.743
		Maintenance	1907	2365.5	18,359.0	497.6	4.8	671.1
	T/F **	Operation	156	196.9	29.6	195.0	1.1	0.309
		Maintenance	372	931.7	924.6	797.6	1.6	12.7
	Overall **	Operation	2833	209.1	121.9	163.4	2.4	0.743
		Maintenance	17,048	638.9	6316.5	250.8	2.8	671.1
Package and test	A/P	Operation	36	6281	2490	43.1	1.2	14.0
		Maintenance	1440	15,730	76,879	45.7	1.6	2230.5
	MDL	Maintenance	3662	4588	5249	31	1.5	59.8
	Test handler	Maintenance	93	11,633	34,887	45.2	1.4	339.1
	Overall **	Operation	36	6280.6	2490.2	43.1	1.2	14.0
		Maintenance	5195	7802.1	41,272.6	34.7	1.6	2230.5

CMP: chemical mechanical polishing, DIFF: diffusion, ETCH: etching, IMP: ion implantation, PHOTO: photolithography, T/F: thin film, A/P: adhesive print, MDL: module, AM: arithmetic mean, SD: standard deviation, GM: geometric mean, GSD: geometric standard deviation, Max: maximum, **: *p* < 0.01 (comparison of concentration between operation and maintenance by *t*-test).

**Table 6:** Results of alarms (*n* = 101) from gas detectors over 10 months from 2014 to 2015.

Classification		N	%
Type of gas ^†^ triggering alarm	TEOS [Si(OC_2_H_5_)_4_]	44	43.6
	Difluoromethane [CH_2_F_2_]	18	17.8
	Hydrogen bromide [HBr]	9	8.9
	Hexafluorobutadiene [C_4_F_6_]	5	5.0
	TMB [B(CH_3_)_3_]	5	5.0
	Diborane [B_2_H_6_]	3	3.0
	Boron trichloride [BCl_3_]	3	3.0
	Others ^††^	14	13.9
Cause of alarm events	Preventive maintenance work using isopropyl alcohol	71	70.3
	Interruption	7	6.9
	Unknown	17	16.8
	Others ^†††^	6	5.9
Level of alarm	>OEL and <2xOEL	62	61.4
	≥2xOEL	39	38.6

^†^ 47 (73.4%) of 64 gaseous chemicals used were monitored through 1486 gas detectors; ^††^ Others: Cl_2_, CH_4_, HCl, AsH_3_, CF_4_, PH_3_, SiH_4_, SiHCl_3_, SiH_2_Cl_2_, TiCl4; ^†††^ Others: alarm events occurred during the operation of alarm test, chamber open, parts replacement; TEOS: tetraethyl orthosilicate, TMB: trimethyl boron, OEL: occupational exposure limit.
